# Supplementary material for: Influence of Subcellular Localization and Functional State on Protein Turnover
Source: Cells. 2021 Jul 10;10(7):1747. doi: 10.3390/cells10071747 (PMC8306977; doi:10.3390/cells10071747)
Supplement: Supplementary file 1 [file cells-10-01747-s001.zip › cells-1218022-send proof Supplementary/cells-1218022-send proof Supplementary.pdf]

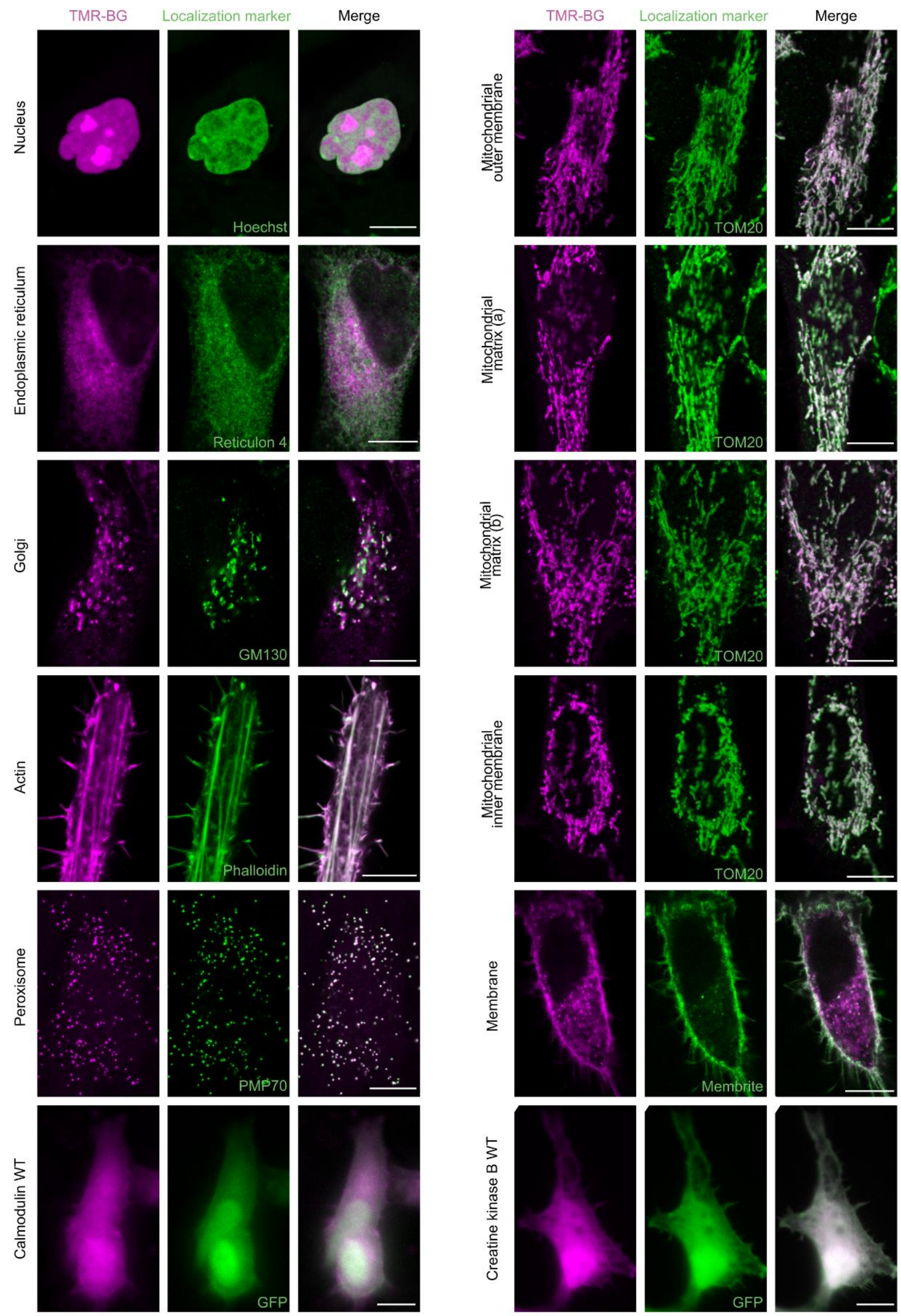

Supplementary Figure 1. Localization of SNAP-tag fusion constructs. The panels show the different SNAP sensors (summarized in Table 1), labelled with TMR-BG (magenta) and the respective colocalization marker (green). Note that NLS in cells is known to be enriched in nucleoli [87], while Hoechst, especially in human cells, is more evenly distributed. Along similar lines, Membrite labelling is a more reliable marker of the plasma membrane, while the palmitoylated-

SNAP sensor is present both on plasma membrane as well as on other membranes within cells, as also previously observed for palmitoylated proteins [88]. Calmodulin WT and Creatine kinase B WT expressing cells were co-transfected with GFP, which is soluble and serves here as a control for soluble protein localization (reproduced from Figure 1). For the localization of the Rab5 sensors, please refer to main Figure 5. Scale bars 10 μm.

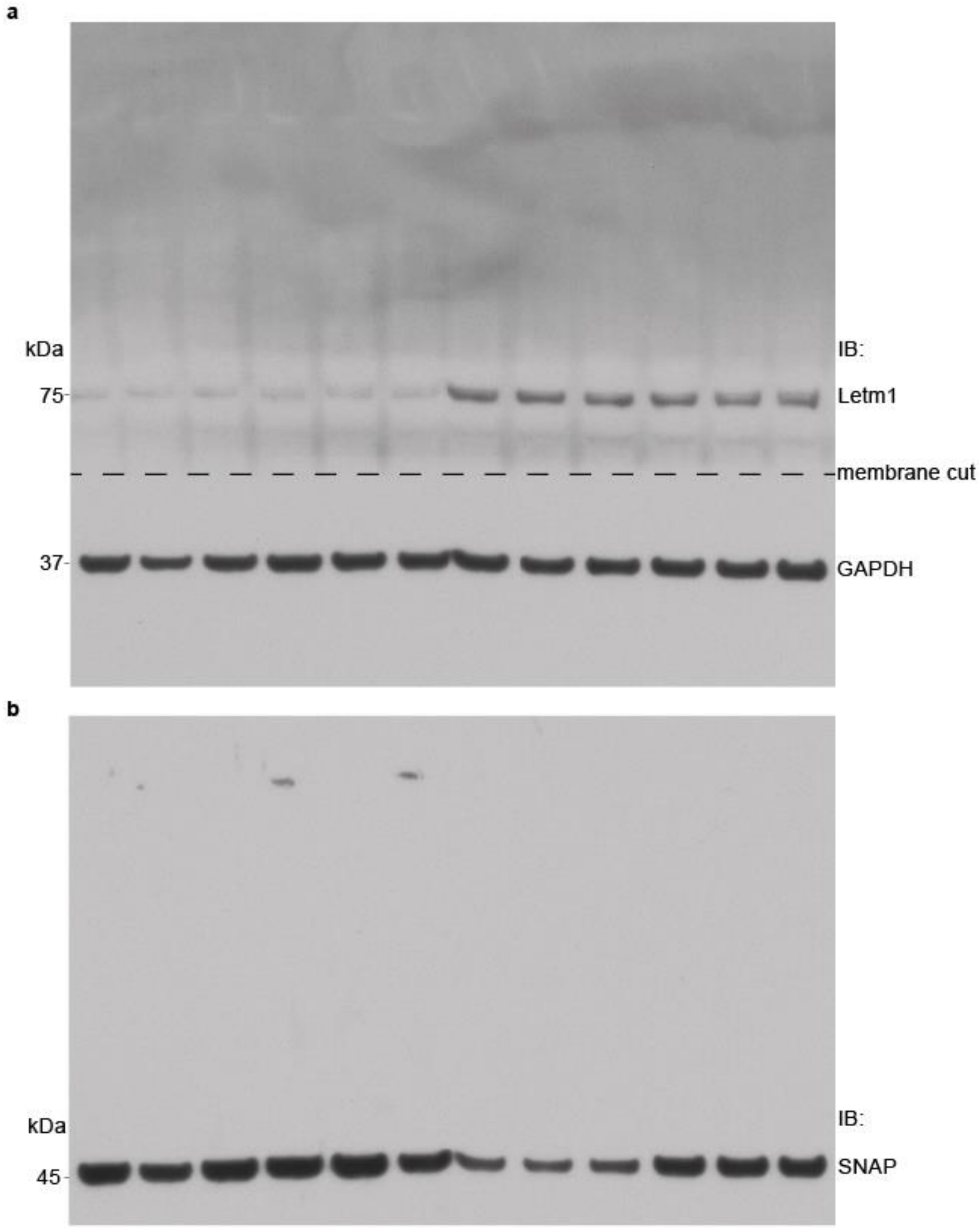

Supplementary Figure 2. Western blot to study Rab5a-WT and Rab5a-Q79L association with the membrane. **a**. Immunoblot for Letm1 (75 kDa) and GAPDH (37 kDa). The position where the membrane was cut (but exposed on the same film) is indicated by a dashed line. **b**. Immunoblot for SNAP-tag (45 kDa).

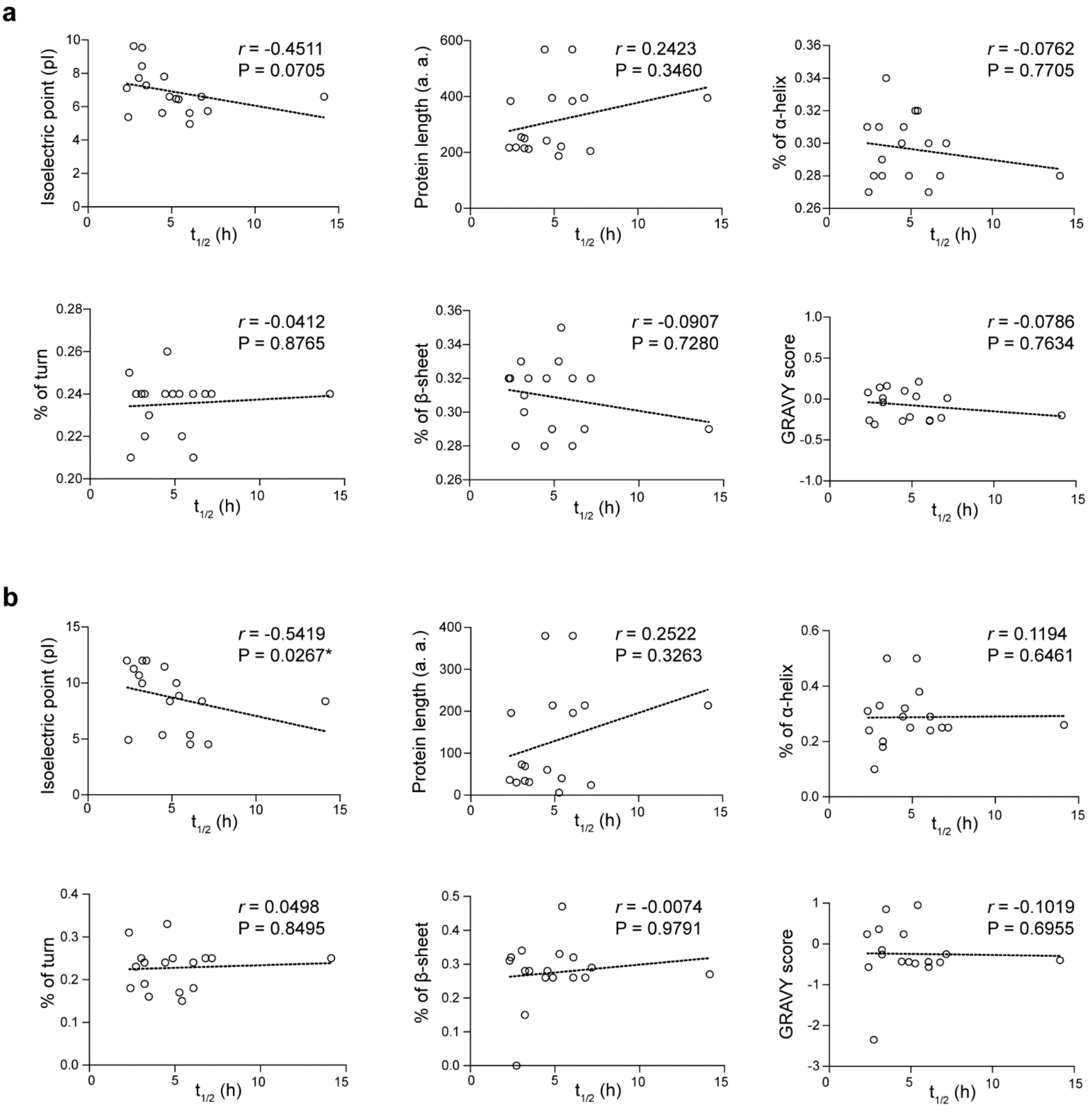

Supplementary Figure 3. Correlations of protein half-lives with biochemical properties including (a) or excluding (b) SNAP-tag. a-b. Correlations between protein half-lives with various characteristics of proteins considering (**a**) or not considering (**b**) the SNAP-tag sequence in the calculation. These measures include the isoelectric point, the protein length, the percentage of alpha-helix, turn or beta-sheet and grand average of hydropathy (GRAVY), a measure of hydrophobicity. See methods for details. All the data are presented as scatter plots with linear fits represented by a segmented line. *r* – Spearman's rank correlation coefficient (N = 17, data does not follow Gaussian distribution).
